# A Homogeneous Label-Free Electrochemical microRNA Biosensor Coupling With G-Triplex/Methylene Blue Complex and λ-Exonuclease-Assisted Recycling Amplification

**DOI:** 10.3389/fchem.2021.753253

**Published:** 2021-11-04

**Authors:** Yao Meng, Fangming Chen, Mingrui Jiang, Qin Guo, Yaqiong Wang, Jian Wang, De-Wen Zhang

**Affiliations:** ^1^ Department of Biophysics, School of Basic Medical Sciences, Institute of Medical Engineering, Health Science Center, Xi’an Jiaotong University, Xi’an, China; ^2^ Key Laboratory of Environment and Genes Related to Diseases, Xi’an Jiaotong University, Ministry of Education of China, Xi’an, China

**Keywords:** homogeneous label-free, microRNA, functional nucleic acid, signal amplification, electrochemical biosensor

## Abstract

A novel homogeneous label-free electrochemical biosensor using G-triplex/methylene blue (G3/MB) complex as the signal generator together with an amplification assisted by the λ-exonuclease (λ-Exo) has been successfully constructed for ultrasensitive microRNA (miRNA) detection. An integrated microelectrode was designed to realize the miniaturization of the homogeneous electrochemical assay. Taking advantage of G3, that can specifically bind with MB and decrease its diffusion current, a single-stranded functional DNA hairpin structure was designed as the bio-recognition probe. The probe consisted of G3, eight bases to block G3, and the complementary sequences of the target miRNA. Here we chose miRNA141—a potentially diagnostic biomarker of prostate cancer as the model target. The presence of miRNA141 could hybridize with the probe DNA to form a double-stranded structure with a 5′-phosphorylated terminus. Then λ-Exo was adopted to digest mononucleotides from the 5′-end, leading to the release of G3 part and miRNA141. The released miRNA could hybridize with another probe to trigger the cycling process, while the released G3 could therefore interact with MB to cause a detectable decrease of diffusion current. The proposed strategy showed a low detection limit of 16 fM and an excellent specificity to discriminate single-base mismatches. Furthermore, this sensor was applied to detect miRNA141 from diluted human serum samples, indicating that it has great potential in the application of nucleic acid detection in real samples.

## Introduction

Electrochemical biosensing based on functional nucleic acid is currently a research hotspot in the field of biochemical analysis and detection. It combines the advantages of simplicity, rapidity, low consumption, and high sensitivity of electrochemical biosensors (Maduraiveeran et al., 2018; Wu et al., 2021b) with the high specificity of functional nucleic acids (Hai et al., 2020). At present, functional nucleic acid-based electrochemical biosensors are mainly constructed using heterogeneous methods (Trotter et al., 2020), which are highly sensitive with low consumption. However, heterogeneous detections usually involve chemical or physical modifications of the electrode surface with bio-recognition probes, which are not beneficial for the reproducibility and stability (Fu et al., 2013; Li et al., 2017; Zhu et al., 2020). Therefore, how to develop a homogenous electrochemical biosensor based on functional nucleic acid for rapid on-site detection is an urgent problem in current research (Liu et al., 2020).

Homogeneous electrochemical biosensors do not require surface modification, which could not only simplify the operating procedure, but also avoid the steric hindrance effect, thus enhancing the identification and response efficiency (Liu et al., 2017a; Chang et al., 2019). To implement the homogeneous detection in real biosensing applications, there are three issues need to be solved. Firstly, the sample consumption for each disposable reaction should be kept at microliter level or less. Secondly, to thoroughly retain the biological activity of biomolecules and further simplify the preparation process, the development of a label-free homogeneous electrochemical biosensor has become more and more attractive (Chang et al., 2019). Lastly, since most of the homogenous electrochemical detection suffered from low sensitivity due to the measurement of diffusion currents, the combination with signal amplification strategy is of significant importance.

The usage of ultramicroelectrodes provides the possibility for the construction of small-volume consumed homogeneous electrochemical biosensors. The previous work reported a miniaturized device using a carbon fiber ultramicroelectrode assembled into a micropipet tip as the working electrode (Zhang et al., 2013a; Zhang et al., 2013b). Even though the electrochemical reaction was confined in the micropipet tip, making each sample consumption affordable, the three-electrode system still needed milliliter-level supporting electrolyte to place the reference electrode and counter electrode. Consequently, substances in the test solution in micropipet tip would inevitably diffuse into the outside supporting electrolyte solution, resulting in a slow change in their concentrations, which limited the detection time and application scope of the device. In this work, we successfully developed an integrated microelectrode by integrating three electrodes in a triple tube to form a miniaturized three-electrode system. With this improvement, the electrochemical testing process is no longer affected by the diffusion effect. Furthermore, the setup for homogeneous detection becomes much more compact and miniaturized compared to the traditional device.

In label-free electrochemical biosensing, G-quadruplex DNA (G4) is one of the mostly used functional nucleic acid to work as a signal readout element. For example, the hemin/G4, a known horseradish peroxidase mimicking DNAzyme, has been widely used in the construction of various electrochemical biosensors (Wu et al., 2017; Liu et al., 2017b; Chen et al., 2018; Sun et al., 2018; Ji et al., 2019; Wu et al., 2021a). The discovery that the combination of G4 and MB can cause a great reduction in MB diffusion current has further expanded its biosensing application scope (Zhang et al., 2014; Cao et al., 2017; Zhao et al., 2020b). Subsequently, it was found that G-triplex (G3), as a folding intermediate of G4, has an even stronger affinity to MB than G4. The high affinity was demonstrated using circular dichroism and electrospray ionization mass spectrometry. Furthermore, molecular dynamics simulations indicated that intercalation was the major mode of interaction between G3 and MB (Zhao et al., 2019). Since the first report, the G3/MB composite has been proved to be an universal, simple, sensitive and efficient homogeneous signal readout element for electrochemical biosensors (Zhao et al., 2020a; Chen et al., 2021).

Increasing evidences have suggested that a large number of genetic diseases are asso-ciated with miRNAs dysregulation, and miRNA expressions are closely associated with the pathogenesis of most human malignancies (Lu et al., 2018; Zhang et al., 2018). Thus, miRNAs possess the potential to be employed as valuable biomarkers for molecular diagnosis of tumors (Yazdanparast et al., 2020). As a cancer-specific biomarker, the detection of miRNA is of great significance for biomedical research, early clinical diagnosis, disease pathogenesis and therapeutic intervention (Shao et al., 2018; Liu et al., 2019). MiRNAs show ultralow abundance (<1.0 pM) in normal human serum and are often with similar sequences (Wang et al., 2016). What’s more, their concentrations fluctuate markedly during the disease diagnosis (Xu et al., 2019). Therefore, it is very essential to exploit miRNA biosensing strategies with high sensitivity and selectivity. Various amplification methods based on functional nucleic acid such as rolling circle amplification (Zhou et al., 2010), hybrid chain reaction (Ge et al., 2020), and catalytic hairpin assembly (He et al., 2019) have been designed to improve the sensitivity and specificity of miRNA analysis. λ-Exo is a sequence-independent enzyme that does not require a specific recognition site. It can catalyze the stepwise removal of mononucleotides from 5′-terminus of double-stranded nucleic acid. Recently, λ-Exo-assisted target recycling method has been established as a highly effective way for amplified nucleic acid detection (Song et al., 2020).

**Scheme 1:**
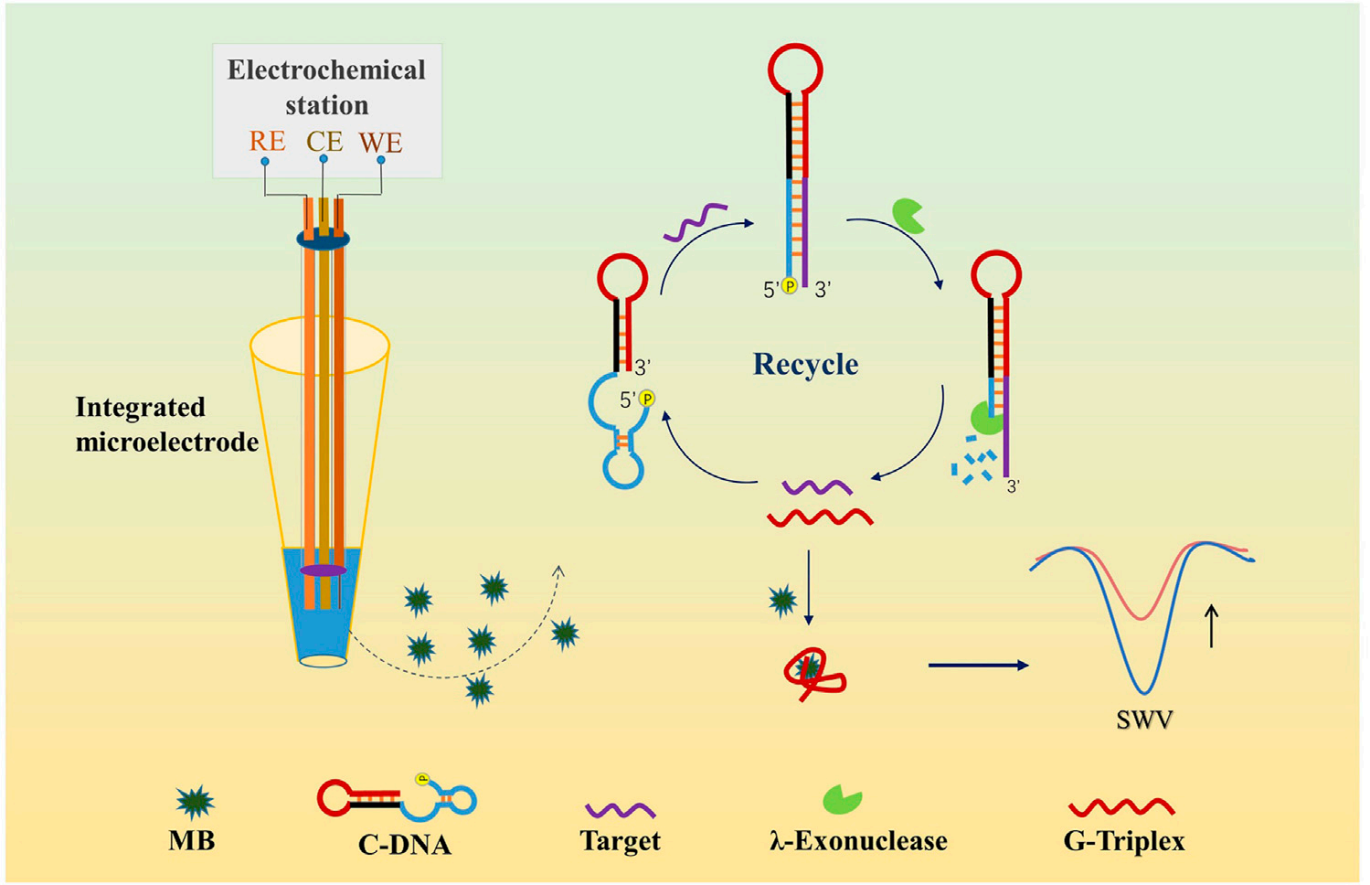
Principle of the label-free homogeneous detection of miRNA.

Herein, inspired by the above findings, we proposed a novel homogeneous label-free electrochemical biosensor for ultrasensitive miRNA detection using miRNA141 as the proof-of-concept analyte. The homogenous disposable detection was realized by using a home-made integrated microelectrode worked at microliter-sample level. G3/MB complex was adopted as the signal generator, coupling with λ-Exo-assisted recycling amplification (Scheme 1). The proposed strategy showed a low detection limit and an excellent specificity to discriminate single-base mismatches. Moreover, the established biosensor was successfully applied to detect miRNA141 from real samples.

## Experimental

### Reagents and Materials

All oligonucleotides were synthesized by Sangon Biological Engineering Technology and Services Co., Ltd. (Shanghai, China) and were stored at −20°C before usage. The detailed sequences are listed in Table 1. λ-Exo was purchased from Harbin Xinhai Gene Testing Co., Ltd. MB was purchased from Kermel (Tianjin, China). K_3_Fe(CN)_6_, K_4_Fe(CN)_6_, KCl, and Tris base were purchased from the Sinopharm Chemical Reagent Co., Ltd. (Shanghai, China), 5× loading buffer for prestained DNA marker was purchased from Generay Biotech Co., Ltd. (Shanghai, China). All oligonucleotides and chemicals were dissolved in 10 mM pH 7.4 Tris-HCl buffer containing 0.1 M KCl. All reagents were analytical reagents and prepared in ultrapure water obtained from a Milli-Q water purification system (Bedford, MA).

**TABLE 1:** All DNA and miRNA sequences used in the work (from 5′ to 3′).

Names	Sequences (from 5′ to 3′)
C-DNA	5’P-CCATCTTTACCAGACAGTGTTA
	*TCC​CTC​CC*CTG​GGA​GGG​AGG​GA
Target miRNA141	UAACACUGUCUGGUAAAGAUGG
Single-base mismatch target (1MT)	UAA​CACCGUC​UGG​UAA​AGA​UGG
Two-base mismatch target (2MT)	UAA​CACCGUCCGGU​AAA​GAU​GG
Three-base mismatch target (3MT)	UAA​CACCGUCCGGCAAA​GAU​GG

In C-DNA, the bold bases are the complementary sequence of the target, the italic bases are the bases that block G3, and the normal bases are the G3 sequence. Underline portion represents mismatched base in target miRNA.

### Fabrication of Integrated Microelectrode

Carbon fibers (7 μm diameter) purchased from Goodfellow company (United Kindom) were cleaned in an ultrasonic bath with acetone, ethanol and ultrapure water for 10 min respectively and dried in air. The carbon fiber (∼1 cm length) connected to a copper wire (0.3 cm diameter, 6 cm length) through conductive silver paint was used as the working electrode (Chen et al., 2021). A carbon rod (0.4 cm diameter, 6 cm length) was used as the counter electrode. An Ag/AgCl reference electrode was prepared by electroplating AgCl on a silver wire (0.3 cm diameter, 6 cm length) in 0.1 M HCl solution with the chronopotentiometry at 0.057 mA for 45 min. Triple tube integrated with three glass microtubes (outer diameter 0.7 cm, inner diameter 0.5 cm), purchased from Nanjing Geolege Technology Co., Ltd., was applied to place the three electrodes, respectively, ensuring a compact structure without physical contact between the electrodes. One end of the triple tube with carbon fiber was sealed with silicone rubber, and the other end was sealed with epoxy resin. Before use, the prepared integrated electrode was quickly swept over the outer flame of the alcohol lamp for cleaning of the carbon fiber (Zhang et al., 2013a). A scanning electron microscope (GeminiSEM 500, Germany) was used to observe the morphology of the tip of the prepared electrode.

### Electrochemical Measurements

All electrochemical measurements were carried out in the home-made miniaturized integrated three-electrode system with a CHI 660E electrochemical workstation (Shanghai Chenhua Instruments Co., Shanghai, China). The schematic diagram of the detection system was shown in Scheme 1. The cyclic voltammetry (CV) was measured at a scan range of −0.6 ∼ 0.6 V and a scan rate of 0.1 V/s. The Square wave voltammetry (SWV) was recorded at a frequency of 200 Hz, a pulse amplitude of 25 mV with an incremental potential of 4 mV. Before electrochemical measurements, C-DNA and miRNA dissolved in Tris-HCl buffer solution were annealed using TC1000-G Gradient PCR (DLAB, Beijing, China). The annealing process consisted of heating the sample to 95 °C for 5 min, and then slowly cooling it to 25°C at 0.05°C/s. The recycling amplification reaction was carried out by mixing 5 μL λ-Exo (2 U/uL), 5 μL MB (10 μM), 5 μL C-DNA (10 μM), and 5 μL target miRNA (100 fM ∼ 1 nM) to a final volume of 20 μL, followed with 40 min’ incubation at 25°C. 40 min was the optimal incubation time as at this time a highest target-induced current drop was obtained. Afterward, the mixed solution was sucked into the pipette tip and then the tip was assembled with the prepared integrated microelectrode. All electrochemical measurements were performed at room temperature.

### Native Polyacrylamide Gel Electrophoresis

Native polyacrylamide gel electrophoresis (PAGE) was performed on gel electrophoresis apparatus (BioRad, Singapore) to demonstrate the reaction between oligonucleotides. Marker (25 ∼ 500 bp) was purchased from BBI Co., Ltd. Firstly, the polyacrylamide gel was prepared from a mixed solution containing 20% acrylamide, 0.07% ammonium persulfate, 0.035% tetramethylethylene diamine, 20% 5 × TBE buffer, and ultrapure water. Then, four samples (C-DNA, miRNA141, C-DNA/miRNA141, and C-DNA/miRNA141/λ-Exo) were incubated at 25°C for 40 min. The concentrations of nucleic acids were all 200 nM and the λ-Exo was 2 U/μL. Next, 8 μL sample mixed with 2 μL loading buffer was subjected to a 20% native PAGE. Electrophoresis was performed in 1 × TBE buffer at 110 V for 2 h at room temperature. After Gel-Red staining, gels were scanned using ChemiDoc^™^ XRS + System (BioRad).

## Results and Discussion

### Design of the Electrochemical Biosensor

The detection principle for miRNA was shown in Scheme 1. In this assay, we designed a single-stranded functional DNA hairpin probe (C-DNA) including three parts: The G3 sequence (in red color), the eight bases to block G3 (in black color), and the complementary sequence of the target miRNA with a 5′-phosphorylated terminus (in blue color). The secondary structure and free energy of the hairpin were evaluated by the OligoAnalyzer program (Integrated DNA Technologies, Inc., United States). Eight was the optimal base pair number by calculating the free energy of the functional nucleic acid as −7.99 kcal/mol. The design of the DNA hairpin structure has strong universality as it can be used to detect various target DNA or RNA just *via* adjusting the complementary sequences. In this work we chose miRNA141 as the model target. With the presence of miRNA141, the hybridization with C-DNA could form a double-stranded nucleic acid with a 5′-phosphorylated terminus, which was then recognized and digested by λ-Exo. Since only the complementary sequence and the blocked bases could be digested, the G3 and the target miRNA were released. The released target was able to be “recycled” and hybridize with C-DNA to start a new cycle of digestion. Meanwhile, the released G3 was bound to MB, causing a reduction of the diffusion current of MB on the electrode surface. Therefore, an amplified strategy for the detection of low-quantity miRNA was established, which has great potential to promote the development of homogeneous label-free electrochemical biosensing.

### Characterization of the Integrated Microelectrode

**FIGURE 1:**
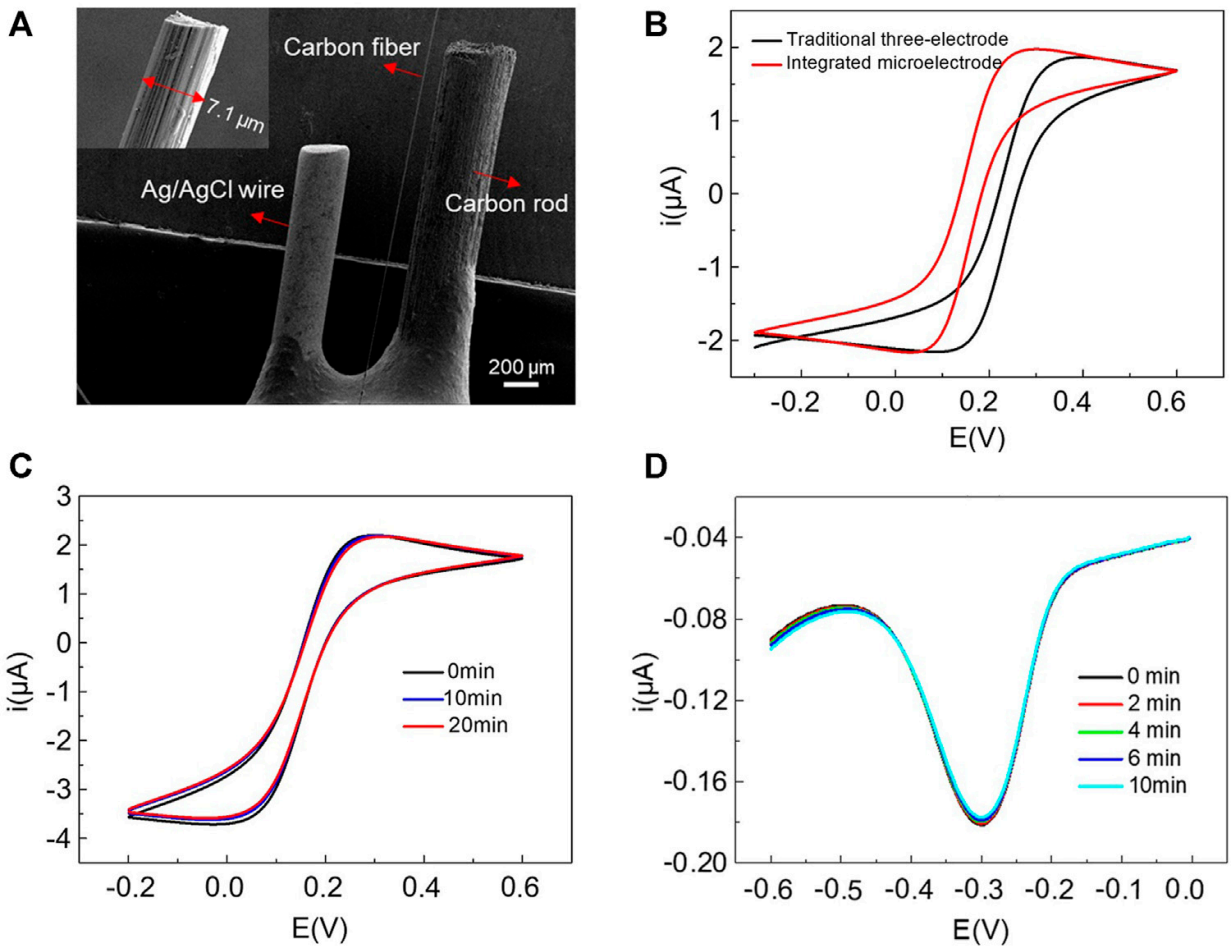
**(A)** SEM images of the home-made integrated microelectrode and the carbon fiber part with a diameter of 7 μm (inset). **(B)** The comparison of CV curves measured with integrated microelectrode and traditional three-electrode system in 5 mM K_3_Fe (CN)_6_/K_4_Fe (CN)_6_ solution. Continuous scanning of CV and SWV using integrated microelectrode in a pipette tip containing 20 μL 5 mM K_3_Fe (CN)_6_/K_4_Fe (CN)_6_ **(C)** and 10 μM MB solution **(D)**, respectively.

The SEM images of the prepared integrated microelectrode and the carbon fiber part were shown in Figure 1A and the inset, respectively. We can see that there was no physical contact between the three electrodes, ensuring a reliable electrochemical measurement. In the inset, the carbon fiber as the working electrode was about 7 μm in diameter. To prove the feasibility of the integrated microelectrode for electrochemical measurements, the CV curves measured with the integrated microelectrode and the traditional three-electrode system in 5 mM K3Fe (CN)6/K4Fe (CN)6 solution were compared. The newly designed electrode was inserted into a micropipet tip filled with 20 μL detection solution, while the traditional three-electrode system was performed in 5 ml solution, using carbon fiber part of integrated microelectrode as the working electrode, an Ag/AgCl reference electrode and a carbon rod counter electrode. The results (Figure 1B) showed a similar curve shape and the same quasi-steady-state current with 70 mV voltage shift, which was due to the use of quasi reference electrode. It indicated that the integrated microelectrode had a similar electrochemical performance to the three-electrode system but measured at a microliter-volume level. The stability of the integrated electrode was characterized by continuously scanning CV curves in a pipette tip containing 20 μL of 5 mM K_3_Fe (CN)_6_/K_4_Fe(CN)_6_ solution (Figure 1C). After 20 min, the curves showed very small changes. The SWV measurements in 10 μM MB solution also showed negligible fluctuations within 10 min (Figure 1D). The above results suggested that this integrated microelectrode has good feasibility and stability, and can be well applied to miniaturized electrochemical detections.

### Feasibility of the Analytical Strategy

**FIGURE 2:**
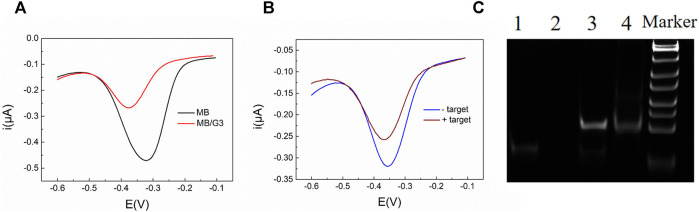
**(A)** SWV response curves of 10 μM MB and 10 μM MB mixed with 10 μM G3; **(B)** SWV response curves of MB (10 μM)/C-DNA (10 μM)/λ-Exo (2 U/μL) and MB (10 μM)/C-DNA (10 μM)/λ-Exo (2 U/μL)/miRNA141 (1 nM). **(C)** Polyacrylamide gel electrophoresis to characterize oligonucleotides in different conditions (lane 1: C-DNA, lane 2: miRNA141, lane 3: C-DNA/miRNA141, lane 4: C-DNA/miRNA141/λ-Exo). The concentrations of nucleic acids were all 200 nM and λ-Exo was 2 U/μL.

Previous experiments have shown that a higher concentration of G3 resulted in a lower current of MB, and the best ratio of G3 and MB was 1:1 (Zhao et al., 2020a; Chen et al., 2021). Therefore, in this experiment, we used the same concentration of 10 μM for MB, G3, and C-DNA. As shown in Figure 2A, 10 μM MB had a high reduction current (curve a). When G3 was mixed with MB at 1:1, the current signal was reduced by 56.5% (curve b), which was consistent with the published result (Zhao et al., 2020a; Chen et al., 2021). The current drop was because G3 was bound to MB to form a G3/MB complex, which could significantly decrease the diffusion rate and inhibit the reduction of MB at electrodes. It verified that G3/MB can be used as an effective signal reporting component due to the obvious drop of diffusion current after binding. Then we demonstrated the assumption that the target miRNA combined with λ-Exo can trigger a conformational change and selective digestion of the hairpin probe to release G3 to produce a current drop. As expected, the presence of miRNA141 obviously decreased the current signal of MB (Figure 2B), indicating the success release of G3 in C-DNA under the synergistic action of the target and λ-Exo. Next, polyacrylamide gel electrophoresis (PAGE) was performed to further validate the reaction. As the results shown in Figure 2C, with the same concentration, the C-DNA exhibited a band (lane 1), while the miRNA141 band was almost invisible (lane 2). It is due to the Gel-Red dye used for PAGE is more sensitive to double-stranded structure consisted in the C-DNA hairpin. The mixture of C-DNA and miRNA141 (lane 3) showed a clear new band at the position above the band of C-DNA, indicating the formation of the hybridization product with reduced mobility. As C-DNA and miRNA were not combined completely, a week band corresponding to C-DNA can be still observed in lane 3. With the addition of λ-Exo, the intensity of the lane related to the combination of C-DNA and miRNA decreased significantly (lane 4), suggesting the successful digestion of the hybridization product. Meanwhile, the C-DNA band in lane 4 was almost disappeared due to the continuous consumption of C-DNA during the cyclical process. Thus, both electrochemical measurements and PAGE verified the feasibility of our analytical strategy.

### Sensor Responses Toward miRNA141

**FIGURE 3:**
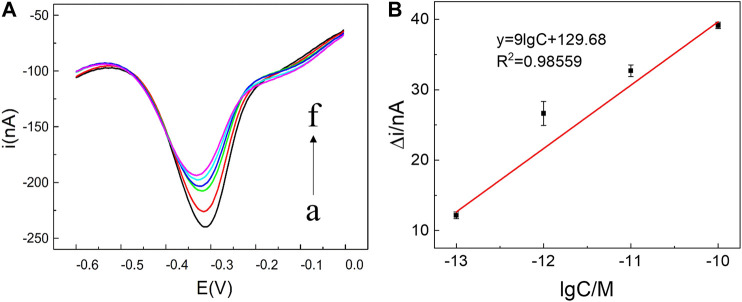
**(A)** SWV current responses of miRNA141 at different concentrations: (a) 0, (b) 100 fM, (c) 1 pM, (d) 10 pM, (e) 100 pM, (f) 1 nM. The recycling amplification reaction was carried out by mixing 5 μL λ-Exo (2 U/uL), 5 μL MB (10 μM), 5 μL C-DNA (10 μM) and 5 μL target miRNA (100 fM ∼ 1 nM) to a final volume of 20 μL. All chemicals were dissolved in 10 mM pH 7.4 Tris-HCl buffer containing 0.1 M KCl. **(B)** Linear relationship between *Δi* and the logarithm of the miRNA141 concentration, in which *Δi* = *i - i*_*0*_ *,* where *i*_*0*_ and *i* were peak currents of SWV in the absence and presence of miRNA-141 (10^−13^ M to 10^−10^ M) respectively. The error bars represented the standard deviation of three measurements.

To inspect the sensor performance of the developed homogeneous label-free miRNA assay, target miRNA141 at different concentrations were incubated with annealed C-DNA, MB, and λ-Exo to trigger the conformation switching and recycling amplification processes. As illustrated in Figure 3, the SWV peak current gradually decreased with the increase of miRNA concentration from 100 fM to 1 nM, which agreed with the fact that a higher concentration of miRNA would induce more G3 release and form more G3/MB complex to reduce the diffusion current. The detection range of the biosensor towards miRNA was determined to be 100 fM ∼ 1 nM. Furthermore, a linear relationship (R^2^ = 0.98559) was obtained versus the logarithm of miRNA141 concentrations in the range from 10^−13^ M to 10^−10^ M (Figure 3B). The limit of detection (LOD) was then estimated to be 16 fM. Compared with the recent detection methods of miRNA141 shown in Table 2, this method takes advantages of a simple fabrication and operating procedures as well as a relatively high sensitivity.

**TABLE 2:** Comparison of this biosensor and other reported methods for miRNA141 detection based on different signal amplification strategies.

Analytical methods	Amplification strategy	Detection rang	Detection of limit	Reference
Photoelectrochemistry	Cascade enzyme-assisted	1 pM ∼ 50 nM	0.2 pM	Zhao et al. (2021)
Surface plasmon resonance	GO-AuNPs hybrids	0 ∼ 50 pM	1 fM	Wang et al. (2016)
Mass spectrometry	Duplex-specific nuclease assisted	0 ∼ 11.25 nM	42 pM	Shi et al. (2019)
Quantitative real-time PCR	Duplex-specific nuclease and strand displacement	1 ∼ 600 pM	10 fM	Liu et al. (2019a)
Fluorescence	Duplex-specific nuclease	1.1 100 nM	100 fM	Yin et al. (2012)
Fiber optic	Optical fiber amplifier	0 ∼ 500 pM	10 pM	Liu et al. (2019b)
Electrochemistry	λ-nuclease assisted	100 fM ∼ 1 nM	16 fM	This work

**FIGURE 4:**
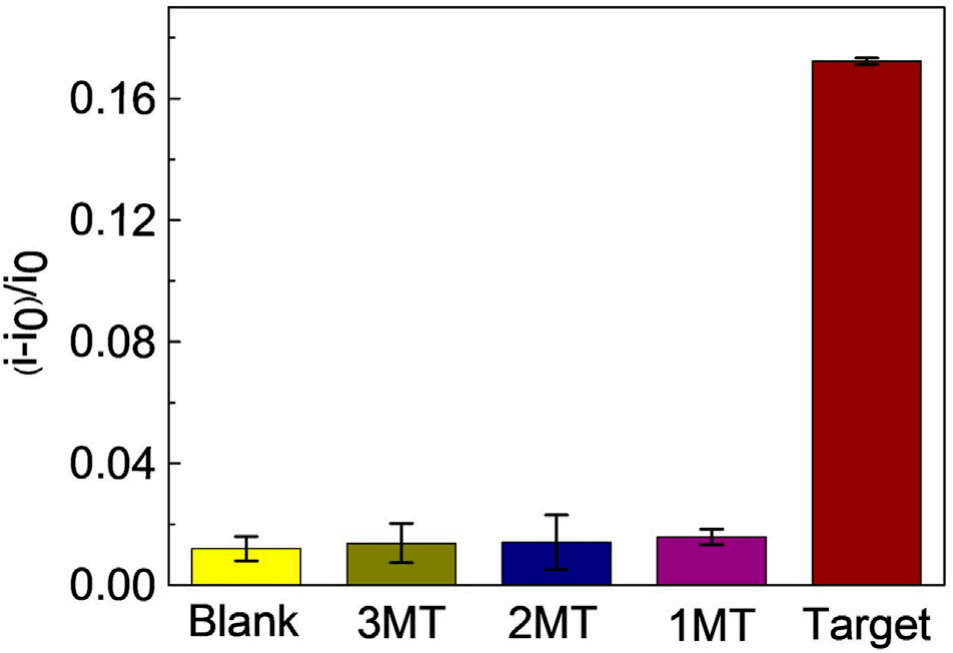
Selectivity of the proposed miRNA biosensor. Reaction mixtures contained 5 μL λ-Exo (2 U/uL), 5 μL MB (10 μM), 5 μL C-DNA (10 μM), and 5 μL miRNA (3MT, 2MT, 1MT and miRNA141). All chemicals were dissolved in 10 mM pH 7.4 Tris-HCl buffer containing 0.1 M KCl. The error bars represented the standard deviation of three repetitive measurements.

**FIGURE 5:**
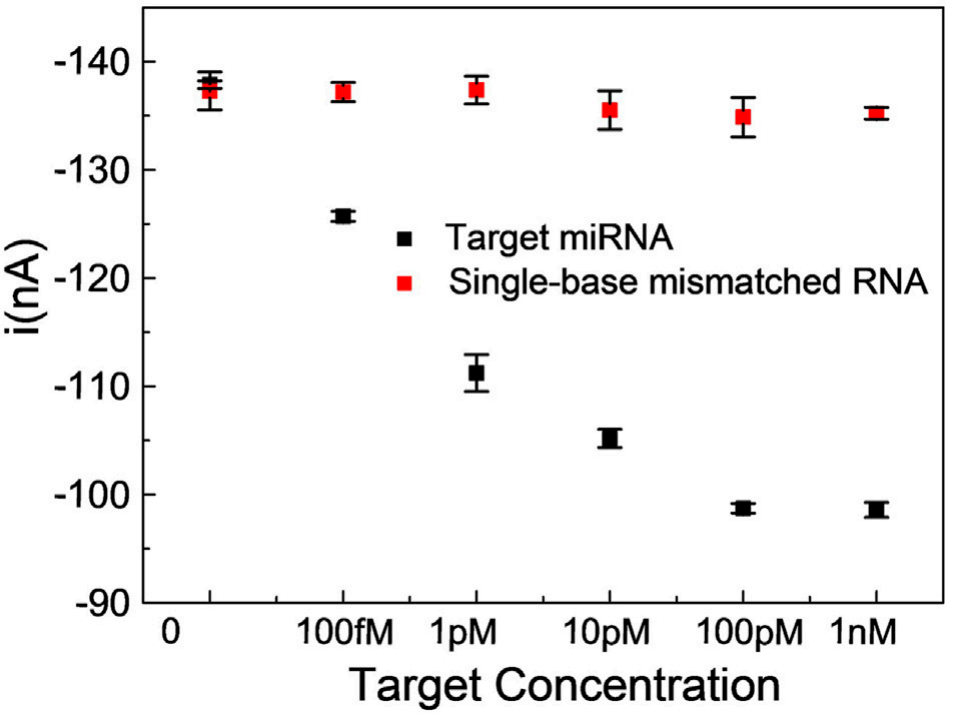
Comparison of SWV peak current values of a single-base mismatched RNA with miRNA141 in the concentration range of 100 fM ∼ 1 nM. Reaction mixtures contained 5 μL λ-Exo (2 U/uL), 5 μL MB (10 μM), 5 μL C-DNA (10 μM), and 5 μL miRNA (target miRNA, 1MT RNA). All chemicals were dissolved in 10 mM pH 7.4 Tris-HCl buffer containing 0.1 M KCl. The error bars represented the standard deviation of three repetitive measurements.

To investigate the specificity of the proposed miRNA biosensor, we compared the responses of the sensor to the target miRNA141 with other three interferences, which were three-base mismatch target (3MT), two-base mismatch target (2MT), and single-base mismatch target (1MT). The results were illustrated in Figure 4. *i* and *i*_*0*_ were peak currents of SWV before and after the addition of miRNA141 or interfering RNA with the same concentration (1 nM). As we can see the three interferers had almost no effect on the diffusion current of MB, while the target miRNA141 significantly reduced the current signal, indicating a good selectivity of the biosensor. Moreover, the dependence of current intensity upon the concentration of miRNA141 and 1 MT RNA were measured under the same condition. A good sensor response to miRNA141 was obtained, while only slight fluctuations of the current to the single-based mismatch RNA were exhibited (see Figure 5). The above results suggested that our proposed assay had an excellent selectivity, which can successfully discriminate one nucleotide variation.

In order to explore the possibility of the biosensor in clinical application, miRNA141 in human serum sample (provided by the First Affiliated Hospital of Xi’an Jiaotong University) was detected and the recovery rate was calculated. Prior assay the human serum was diluted to 1% with Tris buffer. Table 3 showed the sensor responses of 10 pM miRNA141 detected in buffer solution and diluted human serum solution. *Δi*_*buffer*_ and *Δi*_*serum*_ were the changes of SWV peak current after the addition of miRNA141 in Tris buffer and in diluted human serum solution, respectively. As shown in table 3, the recovery rate Δiserum/Δibuffer (%) was from 91.6 to 92.5%, while the relative standard deviation value was in the range of 2.1–3.8%, which suggested that the sensor had the potential to be used in real samples.

**TABLE 3:** The rate of recovery detection of 10 pM miRNA141 in human serum samples.

	Sample	Detection in buffer	Detection in human serum sample	Rate of recovery
C_miRNA141_ = 10 pM		*Δi*_*buffer*_ (nA)	*Δ*iserum (nA)	*Δ*iserum/*Δi*_*buffer*_ (%)
	1	27.6 ± 2.7	25.4 ± 2.3	92.2 ± 2.1
	2	29.6 ± 0.7	27.3 ± 0.6	92.5 ± 3.8
	3	27.6 ± 0.8	25.3 ± 1.1	91.6 ± 2.2

## Conclusion

In summary, we developed a simple and novel homogeneous label-free electrochemical biosensor for highly sensitive and specific detection of target miRNA141. The sensor used G3/MB complex as the signal reporting element, exonuclease-mediated scheme for signal amplification, and a home-made integrated microelectrode for miniaturized homogeneous electrochemical analysis. It takes advantages of the high binding affinity between G3 and MB leading to a significant decrease in diffusion current, as well as the unique function of λ-Exo to preferentially cleave 5′-end phosphorylated double-stranded nucleic acids. By combing these strategies, a competitive detection limit of 16 fM as well as a selectivity to distinguish even single base mismatches have been demonstrated with the presented assay. Furthermore, the integrated microelectrode we designed could successfully reduce the sample volume of electrochemical detection to microliters, thoroughly making the simple, fast, and disposable homogeneous detection possible. This established biosensor has the potential to be a universal method for other DNA or RNA detections by changing the corresponding probe sequence, which is considerable attractive in the application of in-field electrochemical analysis and detection.

## Data Availability

The original contributions presented in the study are included in the article/Supplementary Files, further inquiries can be directed to the corresponding authors.
